# Colocutaneous Fistula after Open Inguinal Hernia Repair

**DOI:** 10.1155/2016/2019212

**Published:** 2016-09-21

**Authors:** Maria Isaia, Demetris Christou, Panayiotis Kallis, Nikolaos Koronakis, Panayiotis Hadjicostas

**Affiliations:** Department of General Surgery, Larnaca General Hospital, Larnaca, Cyprus

## Abstract

The plug-and-patch technique is frequently used for the open repair of inguinal hernias; however, serious complications may arise on rare occasions. We present the case of a 69-year-old patient who presented with a colocutaneous fistula with the sigmoid colon 9 years after the repair of a left sliding inguinal hernia with the plug-and-patch technique. The patient underwent sigmoidectomy and excision of the fistulous track. He was discharged on postoperative day 5 and had an uneventful recovery. Although such complications are reported rarely, the surgeon must be aware of them when deciding upon the method of hernia repair.

## 1. Introduction

Open inguinal hernia repair is probably the most widely performed operation worldwide. Several techniques have been described, each with its own indications and contraindications, but also with its own technical hitches. The surgeons must be aware of all the techniques and possible complications that may arise from these techniques and respond accordingly.

## 2. Case Presentation

A 69-year-old patient presented with a 4-day history of pain and swelling in the left inguinal region. His past surgical history was significant for an open repair of a left sliding indirect inguinal hernia 9 years earlier by the plug-and-patch technique. Ultrasound and Computed Tomography (CT) revealed fluid collection with air in the inguinal area. On operation an abscess was found in the inguinal canal over the previously placed mesh plug, which was drained. The mesh plug was in place and secured with prolene sutures, with no signs of hernia recurrence. The patient was discharged 5 days later on oral antibiotics according to the swab culture which was positive for* Escherichia coli* (*E. coli*).

Two weeks later the patient presented complaining of green coloured discharge from the wound. The cultures were positive for* Pseudomonas aeruginosa* resistant to oral antibiotics and the patient was therefore readmitted for intravenous antibiotic treatment for a period of 7 days. A new culture result showed only* E. coli* sensitive to oral antibiotics and the patient was discharged home.

**Figure 1:**
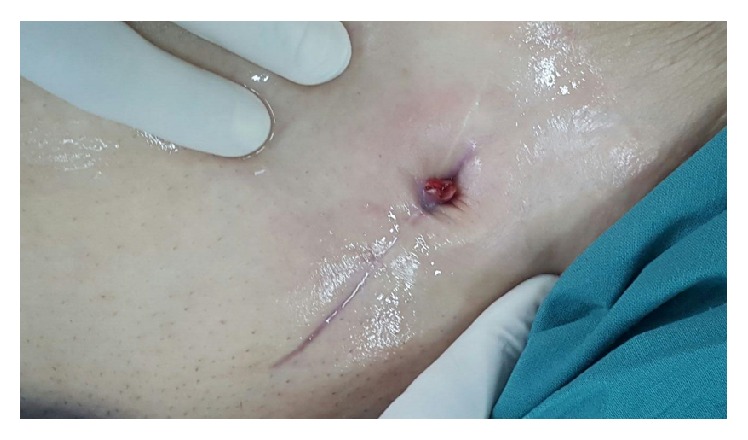
The fistulous track with granulation tissue at the cephalad end of the postoperative wound.

**Figure 2:**
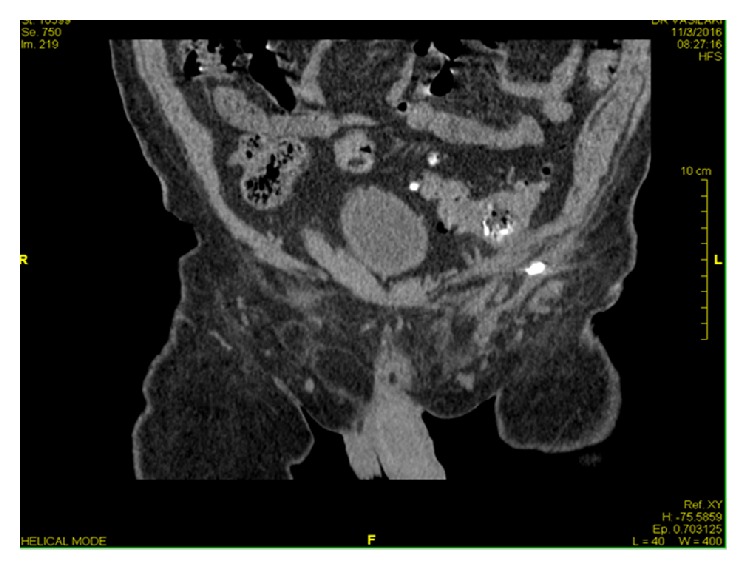
CT scan fistulogram confirming the colocutaneous fistula by the presence of contrast in the colon.

On follow-up 2 weeks later, the wound was not healing and was discharging feculent matter (Figure 1). The patient also reported gas exit from the wound. A CT scan and fistulogram confirmed the presence of a colocutaneous fistula with the sigmoid colon (Figure 2). Colonoscopy revealed the mesh plug protruding into the sigmoid colon at 40 cm from the anal verge and the presence of multiple diverticula.

**Figure 3:**
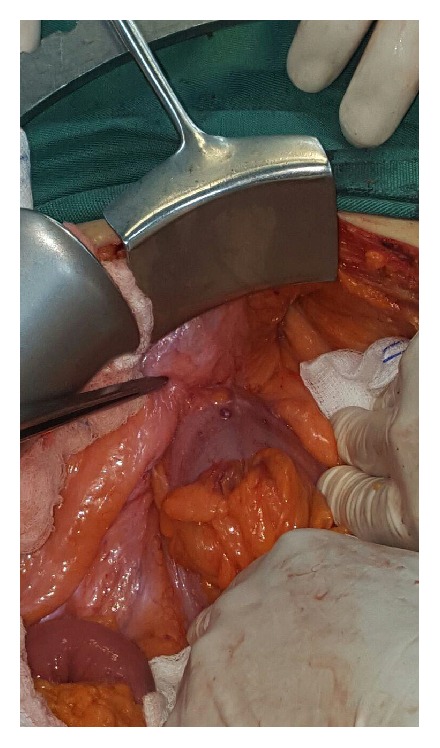
The sigmoid colon strongly adherent to the parietal peritoneum at the level of the internal inguinal ring.

**Figure 4:**
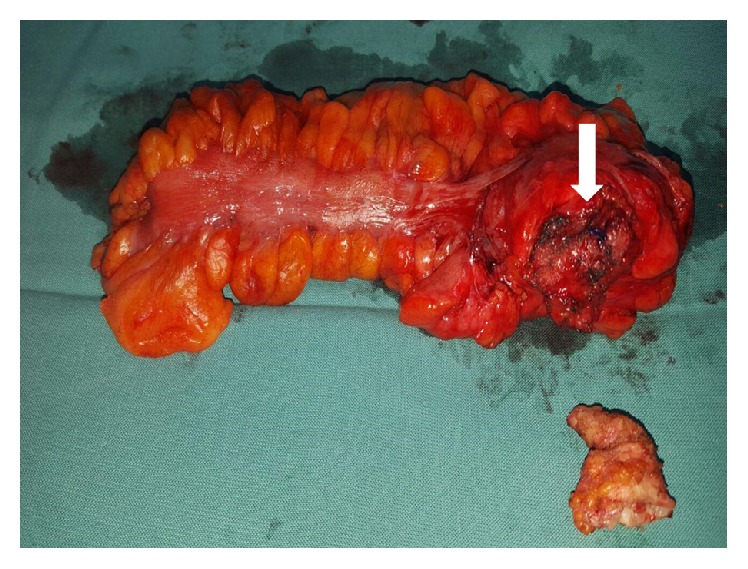
Surgical specimens of the resected sigmoid colon with the mesh plug (arrow) and the fistulous track.

During laparotomy the sigmoid colon was strongly adherent to the parietal peritoneum at the level of the internal inguinal ring (Figure 3). We performed resection of the sigmoid colon along with the mesh plug and excision of the fistulous track from an anterior inguinal approach (Figure 4). The peritoneal defect at the internal ring was suture repaired. The patient was discharged on postoperative day 5 and had an uneventful recovery.

## 3. Discussion

The plug-and-patch technique for open inguinal hernia repair is frequently performed but serious complications may arise on rare occasions. We have presented a case of colocutaneous fistula after open inguinal hernia repair with the plug-and-patch technique. In literature search we have found only 3 cases of colocutaneous fistula with the sigmoid colon after open inguinal hernia repair. In the two cases a mesh plug was used for the repair [1, 2] and the third case was after a Lichtenstein repair [3]. Colocutaneous fistula with the cecum has been reported more rarely [4]. Other cases with plug complications involving the sigmoid colon have been described and presented clinically as intra-abdominal abscesses [5, 6] or sigmoid obstruction [7].

In our case the mesh plug was in place at the internal ring and fixed with prolene sutures but still protruded into the peritoneum and eroded the sigmoid colon. Previous operation notes were available and reported a sliding hernia. In such cases the direct contact between the mesh and colon can lead to pressure necrosis and erosion. A Bard®PerFix*™*Plug was used for the repair. The conical shape of the plug and the fact that it is heavy-weight may have contributed to the erosion.

Another possible explanation of the cause of this complication was diverticulosis. In a case report sigmoid colon diverticulosis was found adherent to a mesh plug [8]. Similar to our case, we suggest that diverticulitis can cause attraction of the plug and subsequent erosion of the colon.

The need of a mesh plug has been questioned in a study concluding that a plug device is not necessary for successful hernia surgery [9]. Another option in order to avoid plug complications may be the use of a bioabsorbable mesh plug [10].

In conclusion, serious complications, although rare, may arise from a simple procedure such as an open inguinal hernia repair. The surgeon must consider them when deciding upon the method of the repair.
